# Inner Ear Gene Therapies Take Off: Current Promises and Future Challenges

**DOI:** 10.3390/jcm9072309

**Published:** 2020-07-21

**Authors:** Sedigheh Delmaghani, Aziz El-Amraoui

**Affiliations:** Progressive Sensory Disorders, Pathophysiology and Therapy Unit, Institut Pasteur, Institut de l’Audition, INSERM-UMRS1120, Sorbonne Université, 63 rue de Charenton, 75012 Paris, France

**Keywords:** sensory disorders, cochlea, sensorineural hearing loss, gene therapy, AAV, genome editing, CRISPR/Cas9, RNAi, antisense oligonucleotide, lipid nanoparticle-mediated delivery

## Abstract

Hearing impairment is the most frequent sensory deficit in humans of all age groups, from children (1/500) to the elderly (more than 50% of the over-75 s). Over 50% of congenital deafness are hereditary in nature. The other major causes of deafness, which also may have genetic predisposition, are aging, acoustic trauma, ototoxic drugs such as aminoglycosides, and noise exposure. Over the last two decades, the study of inherited deafness forms and related animal models has been instrumental in deciphering the molecular, cellular, and physiological mechanisms of disease. However, there is still no curative treatment for sensorineural deafness. Hearing loss is currently palliated by rehabilitation methods: conventional hearing aids, and for more severe forms, cochlear implants. Efforts are continuing to improve these devices to help users to understand speech in noisy environments and to appreciate music. However, neither approach can mediate a full recovery of hearing sensitivity and/or restoration of the native inner ear sensory epithelia. New therapeutic approaches based on gene transfer and gene editing tools are being developed in animal models. In this review, we focus on the successful restoration of auditory and vestibular functions in certain inner ear conditions, paving the way for future clinical applications.

## 1. Introduction

Hearing impairment is the most common sensory deficit in humans [1,2,3]. According to the World Health Organization (WHO), disabling hearing loss affects over 5% of the world’s population (466 million people). About 1 newborn in every 500 suffer from congenital hearing impairment, with over 50% of these being hereditary in nature [4,5,6]. Age-related hearing loss affects almost two-thirds of individuals over the age of 70 years. Environmental factors, such as noise overexposure, viruses, or ototoxic drugs or chemicals, can also result in permanent sensorineural hearing loss, through damage to the auditory hair cells and neurons [1,2,3]. Despite the massive burden posed by hearing problems, current clinical options for treating sensorineural hearing loss are limited, and are mostly based on hearing devices, such as hearing aids and cochlear implants. These treatments are beneficial but cannot restore hearing to normal levels. 

Therapeutic approaches targeting the inner ear are based on an increasingly detailed knowledge of the molecular physiology of inner ear function, and of the biological and molecular mechanisms underlying vestibular and auditory defects. About 140 non-syndromic hearing loss genes have been identified, causing impairments of various degrees of severity and progressivity. These genes encode diverse proteins, with different functions in the inner ear, including gene regulation, ion homeostasis, synaptic transmission, and roles in auditory hair cell bundle morphology and development [7,8]. As in all therapeutic approaches, each avenue explored must be appropriate for (i) the nature of the causal agent and its defective mechanism; (ii) the target cells (auditory hair cells, supporting cells, or neurons); (iii) the degree of hearing loss and its progressivity; and (iv) the objective: preventing hearing loss, protecting or restoring function, or replacing damaged cells. In recent years, a growing number of approaches, including gene replacement (gene supplementation), gene suppression (RNA-based therapeutics), and gene editing, have proved effective in animal models of deafness. We review here the therapeutic strategies in current use, and progress towards the curative treatment of hearing loss, and outline the challenges associated with in vivo gene therapy targeting the human inner ear for the treatment of human sensorineural hearing loss.

## 2. The Inner Ear and Its Auditory Hair Cells Specializing in Mechanoreception

**Figure 1:**
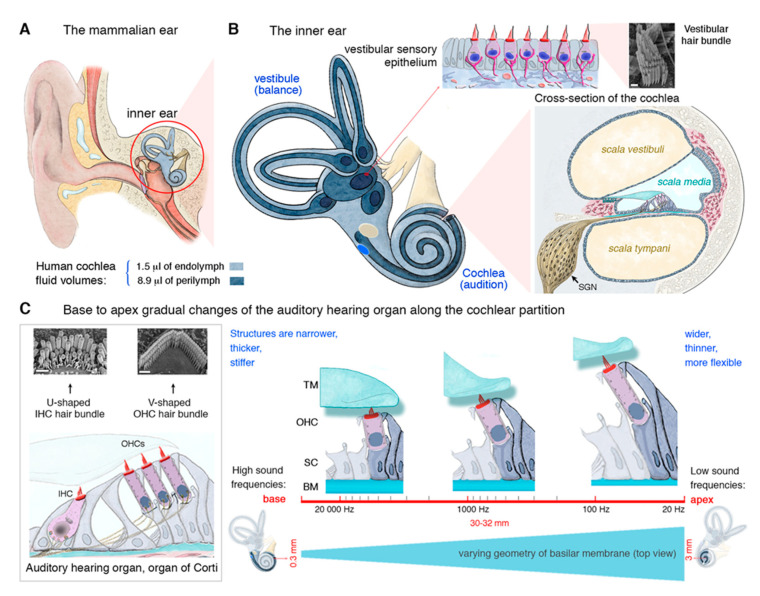
Mammalian inner ear anatomy and cochlear tonotopic organization. The mammalian inner ear consists of the vestibule (balance organs), which detect linear and angular accelerations, and the cochlea, the hearing organ, which detects sound waves. (**A**,**B**) The cochlea is made up of three fluid-filled compartments of differing ionic compositions—*scala vestibuli* (perilymph), the *scala media* (endolymph), and the *scala tympani* (perilymph). Sound conversion into electrical signals requires three major types of functional cells: hair cells (purple), supporting cells, and spiral ganglion neurons (yellow). (**C**) The auditory sensory organ, the organ of Corti, is made up of one row of highly organized inner hair cells (IHCs), three rows of outer hair cells (OHCs), flanked by various types of supporting cells. Along the cochlea, the hair cells, underlying basilar membrane (BM), surrounding, and overlying tectorial membrane (TM) are optimized to perceive specific and characteristic sound frequencies, defining a cochlear tonotopy that is preserved up to the auditory cortex. The structural and physical properties of the cochlea vary from base (shorter and stiffer cells) to apex (longer and more flexible cells). The cochlear base mainly perceives high-frequency tones (up to 20 kHz in humans), while the apex detects low-frequency sounds (20 Hz in humans). Scale bar in B, C: 1 μm.

The mammalian inner ear houses the sensory organ for hearing (the cochlea) and the organ responsible for balance (the vestibule) (Figure 1A,B). The vestibular end organs include the cristae of the three semicircular canals, which respond to angular acceleration, and the maculae of the utricle and saccule, which respond to linear acceleration. Over millions of years of evolution, our ears have developed a highly specialized architecture, with extremely sensitive mechanosensitive equipment in the form of the auditory hair cells. The auditory sensory organ of mammals contains two types of hair cells, so named because they carry a bundle of actin-based stereocilia on their apical surface, responsible for mechanoelectrical transduction: the outer hair cells (OHCs; 9000 to 12,000 cells organized into three rows), which amplify sound stimuli and are unique to mammals, and the inner hair cells (IHCs; a single row of 3000 to 3500 cells), the genuine sensory cells responsible for transmitting sensory information to the central nervous system. The depolarization of these cells leads to the release of a neurotransmitter (glutamate). The resulting activation of primary auditory neurons is then transmitted, via the auditory pathways, to the auditory cortex (Figure 1B).

This complex architecture of the inner ear is essential for the exceptional auditory performance of mammals, in terms of both the sensitivity of hearing and the range of sound intensities and frequencies perceived. The physical, morphological and molecular properties of hair cells vary along the length of the cochlea, such that each hair cell responds to a particular frequency (its characteristic frequency). Together, they form an apicobasal frequency map, known as the tonotopic map, which is essential for the decomposition of complex sounds into their elementary frequency components (pure tones) in the cochlea. In humans, the spectrum of perceptible sound frequencies extends from 20 Hz to 20 kHz. Each frequency is analyzed at a specific site along the length of the cochlea. The base of the cochlea (where the sensory cells, particularly the OHCs, are shorter and more rigid) is dedicated to the analysis of high frequencies (high-pitched sounds), whereas the apex (where the OHCs are longer and more flexible) is dedicated to the analysis of low frequencies (low-pitched sounds) (see Figure 1C).

The high prevalence of hearing loss is partly due to the unexplained disappearance, during the course of evolution, of the capacity to regenerate auditory sensory cells. Indeed, other species, such as fish and amphibians, have retained the ability to produce hair cells throughout their lifetime. In birds, regeneration is not spontaneous, but any damage to the epithelium triggers a replacement of the damaged cells. By contrast, this ability to replace hair cells in the auditory organ disappears after the completion of embryonic development in mammals. Thus, in mammals, including humans, the numbers of hair cells and associated auditory neurons are predetermined before birth. Any subsequent loss of or damage to these cells leads to an irreversible sensory deficit. This is why, regardless of cause—genetic, environmental, or normal aging—hearing loss is often linked to a loss of auditory hair cells and/or the degeneration of their innervation.

## 3. Genetic Hearing Impairment

**Figure 2:**
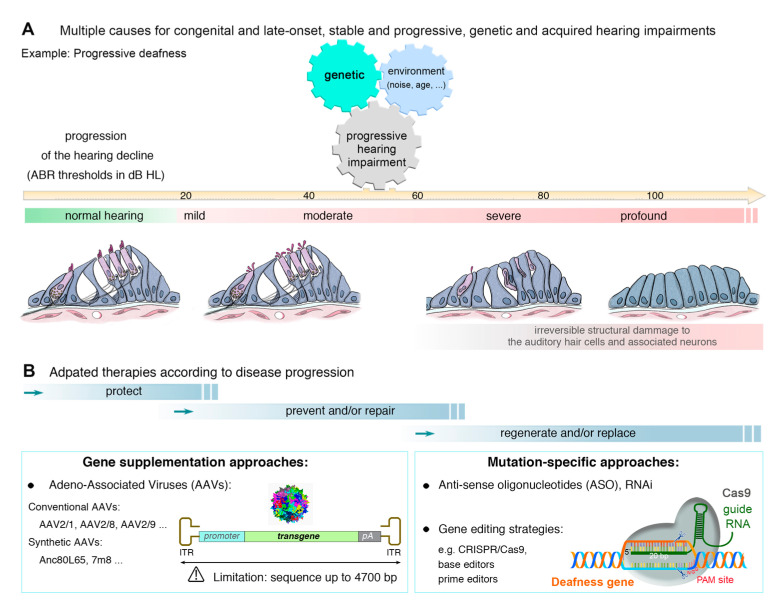
Hearing loss causal origins and adapted therapeutic strategies. (**A**) Hearing loss, defined as mild (loss of 21 to 40 dB HL), moderate (41–70 dB HL loss), severe (71–90 dB HL loss), or profound (>90 dB HL loss), can be due to multiple causes: genetic, noise, and/or age. Whatever the cause, the hearing loss can start any time after birth, with varying degrees of progression and severity. (**B**) Various therapeutic approaches (gene supplementation, silencing, or gene editing) are being implemented either to protect, prevent and/or repair hearing loss, regenerate or replace inner ear cells.

Hearing impairment is the most common form of sensory impairment in humans, affecting one in 500 newborns and around 466 million people worldwide [1]; (http://www.who.int/mediacentre/factsheets/fs300/en/). Environmental factors, such as noise overexposure, aging (Figure 2A), viruses or ototoxic chemicals, can also cause permanent sensorineural hearing loss by damaging auditory hair cells and neurons [1,2,3]. Hearing loss can be classified on the basis of its severity relative to normal hearing, as mild (loss of 21 to 40 decibels of hearing level, dB HL), moderate (loss of 41–70 dB HL), severe (loss of 71–90 dB HL) or profound (loss of >90 dB HL) (Figure 2A). Clinically, hearing impairment may be the only symptom (non-syndromic or isolated hearing loss) or may be associated with other symptoms or abnormalities (syndromic hearing loss). Non-syndromic forms of deafness are classified on the basis of their mode of transmission: autosomal dominant (DFNA), autosomal recessive (DFNB), X chromosome-linked (DFNX), Y chromosome-linked (DFNY), and mitochondrial. DFNB forms account for almost 80% of all cases of prelingual (early-onset) inherited deafness, whereas post-lingual and late-onset forms of hearing impairment generally correspond to DFNA forms.

Most causes of genetic deafness determined to date are monogenic defects. In 1994, the first locus for recessive deafness (DFNB1) was found on 13q12, by linkage analysis in large individual families with isolated deafness [9]. *GJB2* encoding gap-junction protein connexin 26 (Cx26) was subsequently identified as the gene responsible for DFNB1 and DFNA3 [10]. This gene is responsible for a large proportion of the cases of severe-to-profound non-syndromic hearing loss in most populations. The genomic regions linked to hearing loss by linkage analysis and homozygosity mapping often extend over multiple megabases and can contain hundreds of genes. Identification of the gene responsible for deafness from a large number of candidates is an expensive and time-consuming process. Rapid advances in genomics, DNA enrichment, and next-generation sequencing technologies have provided efficient strategies for the selective and cost-effective sequencing of the complete set of coding sequences of the patient (the “whole exome”), accelerating identification of the gene responsible for hearing loss. 

**Figure 3:**
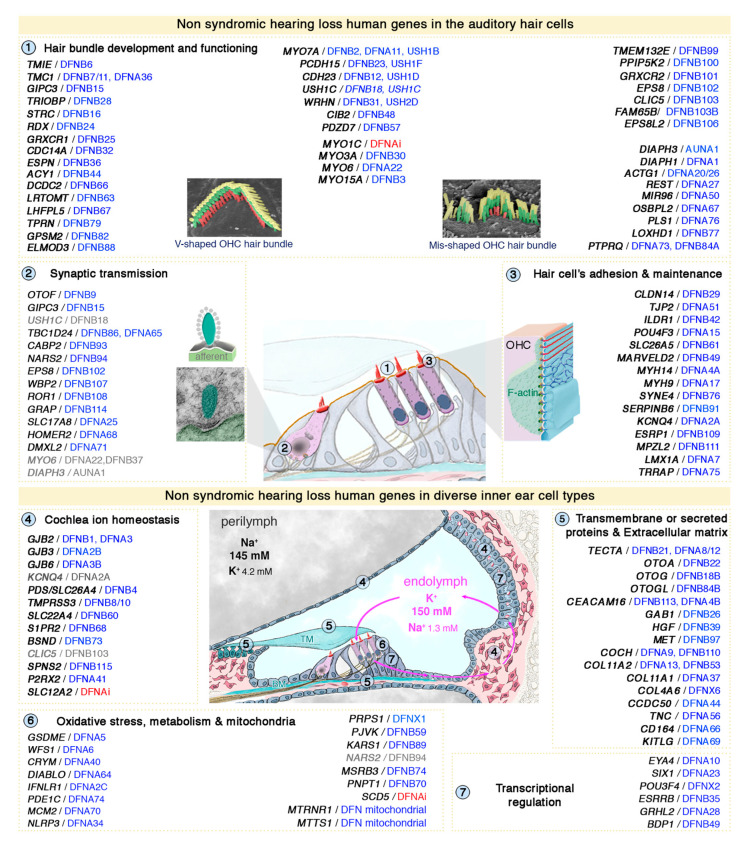
Functional stratification genes/proteins causing human isolated deafness hearing loss. Based on their established role and characterization of corresponding animal models, the human deafness genes (*DFNA DFNB DFNX AUNA*) can be grouped into several functional categories: (1) hair bundle development and functioning, (2) synaptic transmission, (3) hair cell’s adhesion and maintenance, (4) cochlea ion homeostasis, (5) transmembrane or secreted proteins and extracellular matrix, (6) oxidative stress, metabolism and mitochondrial defects, and (7) transcriptional regulation. DFNAi (red) denotes autosomal-dominant forms of deafness with undefined locus number. The genes/loci in grey denote that they share several functional categories. More detailed information regarding the deafness causative genes are provided in Table S1.

Hearing loss is an extremely heterogeneous disorder, with up to about 1,000 different causal genes [11]. About 140 genes have already been shown to cause deafness in humans (see Figure 3), and many more remain to be discovered [12,13,14].

Fortunately, the inner ears and auditory pathways of mice work similarly to those of humans, making it possible to use mouse models to determine the origins of hearing loss [13,14,15,16,17]. In-depth characterization of mouse models of hearing loss and studies of the properties of the predicted protein products of the genes identified have led to the classification of deafness genes into distinct specific subcategories, on the basis of the functions affected: (i) hair bundle development and function, (ii) synaptic transmission, (iii) cell-cell adhesion and maintenance, (iv) ion homeostasis, (v) extracellular matrix, (vi) oxidative stress and mitochondrial defects, (vii) transcriptional regulation (Figure 3). This extensive basic research and improvements in our understanding of the genetic etiology of hearing loss are essential foundations for the development of appropriate treatments for preventing and/or curing cochlear and vestibular disorders.

## 4. Approaches to the Treatment of Hearing Loss

The inner ear is particularly amenable to therapeutic interventions, for two main reasons: (i) the organ is composed of confined compartments to which the therapeutic agent can be delivered with a minimal risk of diffusion beyond the surrounding tissues and (ii) the ear is filled with fluids (endolymph and perilymph), favoring dissemination to a large number of target cells if therapeutic agents, such as viruses, nucleotides and small molecules, are administered locally. The huge diversity of the phenotypic characteristics of deafness, its multiple causes, and the diversity of the target cells involved are key elements that must be considered when designing appropriate treatments. A number of successes, resulting in the correction of hearing and/or balance deficits, have been reported in recent years [2,17,18,19]. We will focus here, in particular, on so-called “gene-specific” approaches aiming to repair or replace the defective gene (Figure 2B). We will also describe strategies targeting pathways common to one or several forms of deafness, which are gene-independent.

### 4.1. Routes for Delivery

**Figure 4:**
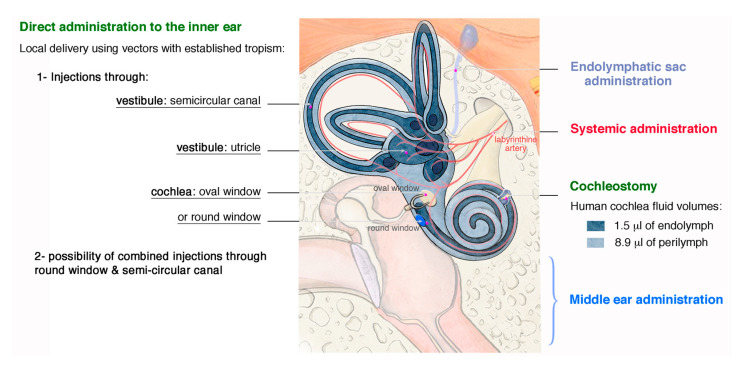
Delivery approaches in the inner ear. Schematic representation of the human ear, illustrating methods used to deliver therapeutics into the inner ear. These include systemic (red) and middle ear (blue) indirect approaches, as well as endolymphatic sac delivery (light blue) and direct injections through different compartments of the inner ear (green): cochleostomy (*scala media*), vestibule (through utricle or semicircular canal), and cochlea (through round window membrane or oval window). Some pros and cons of each methods are highlighted in Table S2.

As explained above, the inner ear has a special architecture, with a fluid-filled spiral cavity set in a bony labyrinth that is hypervulnerable to changes in the quantity and composition of the inner ear fluid. The maintenance of homeostasis following the delivery of therapeutic agents to the inner ear is, therefore, challenging. Three essential routes of delivery to the inner ear have been successfully used in animal models (Figure 4). The most common and successful way of delivering agents to the inner ear is an intracochlear approach, via the round window membrane (RWM), a three-layered membranous opening leading to the perilymphatic space of the scala tympani [20,21,22,23,24,25,26]. This approach is simple and safe, with only a small risk of residual hearing damage. Moreover, György B et al. recently showed that the RWM approach leads to efficient transgene transfer into the cochlea of non-human primates [27]. This approach is, therefore, promising as a means of gene delivery to treat deafness in humans. 

In the second approach, canalostomy, the transgene is injected through a fenestration in the posterior semicircular canal [28,29,30]. The advantage of this method is a wider delivery route through the labyrinth, with no risk of vestibular or auditory damage. This approach may constitute an efficient way of treating some severe forms with both auditory and vestibular dysfunction, as in Usher syndrome. The combination of trans-RWM injection and canal fenestration in adult mice has recently been shown to increase the efficiency of IHC transduction in all turns of the cochlea without impairing auditory function or hearing [31].

Finally, viral injection into the neonatal mouse utricle leads to the transduction of almost 100% of both IHCs and OHCs in the cochlea [32]. By contrast, injections in adult mice result in much lower levels of OHC transduction. Other administration routes have been reported: (i) Systemic application: the use of this route is atraumatic and simple, but it is essential to demonstrate beforehand that the therapeutic agent can cross the blood-labyrinth barrier and reach the target cells in the cochlea. The need for a high dose/titer ratio and volume may be associated with a risk of off-target binding to unwanted tissues or organs; (ii) Middle ear delivery: this includes multiple methods, varying from direct injection of given drug through tympanic membrane or surgical positioning of drug-containing biodegradable compound or delivery device into the middle ear to slowly release doses over time. Permeation enhancers and controlled devices can be used to facilitate local and continuous drug transport to inner ear compartments, in particular through the semi-permeant round window. This procedure is technically simple to perform and generally of low risk, but the concentrations used are generally high and the therapeutic agent must remain in contact with the RWM for a prolonged period to ensure that sufficient amounts cross the RWM; (iii) Cochleostomy: in this approach, the transgene is transferred directly to the scala media, which can be accessed via a hole drilled through the basal part of the cochlea into the cochlear endolymphatic space, near the round window [33,34,35,36,37] (Figure 4 and Table S2). Cochleostomy and RWM injections in adult mice have been shown to have similar efficiencies for the transduction of inner hair cells, but the cochleostomy approach is technically challenging and more likely to results in surgical trauma (as indicated by hearing loss) than the RWM approach [38,39,40].

### 4.2. Gene Therapy Delivery Systems

The success of gene therapy treatments depends on the gene delivery system, more commonly referred to as the “vector,” used to transfer the gene specifically to target cells, and the effective and durable expression of the transgene in target cells. There are two main delivery systems for gene therapy: (i) viral vectors: viruses modified and attenuated to create effective and specific tools for gene transfer, and (ii) non-viral delivery/vectors: nanoparticles and microspheres consisting of biodegradable polymers, such as liposomes (spherical structures formed from a lipid bilayer resembling cell membranes).

#### 4.2.1. Viral Vectors

At least five types of viral vectors have been tested for gene delivery in the ear: retroviruses, lentiviruses [41,42,43,44,45], adenoviruses (AdVs), and adeno-associated viruses (AAVs) [38,46,47,48,49,50,51,52,53,54,55,56], and herpes simplex virus [57,58]. The most promising results to date were obtained with AAVs, which have minimal pathogenic and immunogenic effects [19]. They can genetically modify various non-dividing and dividing cells, to achieve long-term gene expression without integration into the genome [59,60]. AAV is a replication-deficient member of the Parvoviridae family discovered in the 1960s [61]. The AAV genome contains a single DNA molecule flanked by two inverted terminal repeats (ITRs) at either end enabling it to form concatemers in host cells.

At least 12 natural AAV serotypes have been described (AAV1, AAV2, AAV5, AAV6, AAV6.2, AAV7, AAV8, AAV9, rh.8, rh.10, rh.39, and rh.43), and more than 100 variants have been isolated from different animal species [62,63,64,65]. Each serotype has a particular preferential tropism, providing possibilities for improving the cellular targeting of transgene expression. The capacity of each serotype to target a particular type of cell and its transduction efficiency depends on the tissue studied, the dose used, the developmental stage at the time of microinjection and in some cases, the species studied. In the inner ear, AAV1-4, 7, and 8 have been shown to transduce spiral ligament, spiral limbus, and spiral ganglion cells. AAV5 was also shown to be efficient for transducing Claudius cells, sulcus cells, and spiral ganglion neurons [34,38,49,52,66,67]. AAV1-3, 5, 6, and 8 transduce IHCs. AAV1 is the most effective transducer of OHCs and supporting cells [20,22]. Pseudotyped vectors derived from the AAV-2 serotype (AAV2/1, -2/2, -2/5, -2/7, -2/8, and -2/9) have been constructed to increase the potential of AAVs for gene delivery to auditory hair cells and transduction efficiency. All of these vectors displayed tropism for hair cells, but, AAV2/1 was the vector that most efficiently transduced the IHCs and OHCs of the mouse cochlea [42], with AAV2/2 for the most efficient for transducing IHCs in the cochlea of guinea pigs [68]. The use of a specific promoter associated with the transgene, where possible, can provide control over the spatiotemporal pattern of expression of the gene transferred. Most studies have used the human cytomegalovirus immediate-early promoter for transgene expression, but the hybrid CAG promoter sequence from the chicken β—actin promoter and the cytomegalovirus immediate-early enhancer [69,70,71] have provided the most efficient promoter-mediated transgene expression to date in auditory hair cells and neurons.

One major disadvantage of AAVs is their size. Only small genes (up to 4.8 kb) can be incorporated effectively into the vector without a risk of dysfunction (e.g., the production of a truncated protein) [59]. This limitation has driven research on dual-AAV vectors, systems of two AAVs, in which each AAV vector carries a fragment of the larger transgene and the two vectors are reassembled to reconstitute the full-length expression cassette in the target cell [72]. This approach has recently been used to rescue the deafness phenotype in a mouse model of congenital deafness, DFNB9 (*Otof*^−/−^), linked to a defect of otoferlin, a putative Ca^2+^ sensor playing a key role in synapse neurotransmitter release in IHCs [73,74]. The coding sequence of the murine otoferlin cDNA was split into two parts, a 5′ fragment corresponding to the N-terminus followed by a splice donor site, and a donor splice site followed by the Otof 3′ fragment corresponding to the C-terminus of the protein, for reconstruction of the full-length otoferlin from a dual AAV-vector. A single injection of this recombinant AAV vector pair through the round window membrane into the cochlea of *Otof*^−/−^ mice led to a durable restoration of otoferlin expression in transduced inner hair cells, and a total reversal of the deafness phenotype [73].

Another limitation of conventional AAV serotypes in the inner ear is the low transduction efficiency of outer hair cells, resulting in only a partial restoration of hearing. The sequences of capsid proteins have recently been altered to develop and generate novel synthetic AAVs: Anc80L65, AAV9-PHP.B, and AAV2.7m8 [24,25,27,75,76]. Anc80L65 was generated by the in-silico reconstruction of ancestral AAVs [77]. Anc80L65 injections through round window membrane transduce both the cochlear and vestibular sensory organs. This vector is therefore potentially very useful for gene delivery in genetic forms of deafness associated with vestibular dysfunction. Moreover, this vector transduces both inner hair cells and outer hair cells at sufficiently high rates to restore auditory function. In a mouse model of USH1C (due to a defect of the gene encoding harmonin), *Ush1c* c.216G>A, resulting in both auditory and vestibular defects, early postnatal round window membrane injections of AAV2/Anc80L65 encoding harmonin successfully restored auditory and vestibular function to near wild-type levels, particularly at low frequencies (5.6–16 kHz). Structural analysis revealed lower levels of auditory hair cell loss and the preservation of hair bundles with the normal “staircase” morphology in treated ears [25]. In another mouse model for DFNB7/11 recessive deafness, in which the Tmc1 (transmembrane channel-like 1) gene is defective, round window membrane injections of synthetic AAV2/Anc80L65 encoding Tmc1 led to the almost complete restoration of auditory and vestibular function, and morphological rescue [75]. 

AAV9-PHP.B is an AAV9 capsid variant (a randomized heptamer peptide library inserted in the AAV9 capsid), PHP.B, that was originally selected for its efficient broad transduction of mouse neurons within the central nervous system [78]. AAV9-PHP.B injection through RWM transduces the majority of IHCs and about half the OHCs in mice. This vector also transduces retinal photoreceptors and can thus be used to treat Usher 3A patients, who display late-onset blindness, due to retinal degeneration. In a mouse model of Usher syndrome type 3A deafness, *Clrn1*^−/−^ mice, neonatal RWM injections of AAV9-PHP.B-mediated Clrn1 expression under the control of the CBA promoter, resulting in robust hearing rescue (an increase in sensitivity of up to 50 dB), especially at lower frequencies (4–8 kHz) [27]. Little or no rescue was achieved at higher frequencies, possibly due to the relatively low levels of AAV9-PHP.B-mediated Clrn1 expression at the base of the cochlea. Interestingly, the RWM injection of this vector in juvenile cynomolgous monkeys resulted in the highly efficient transduction of both IHCs and OHCs [27]. Interestingly, Ivanchenko et al. showed that higher dose injection of AAV9-PHP.B in juvenile cynomolgous monkeys transduced nearly 100% of both IHCs and OHCs, from base to apex [79]. This vector is, thus, a promising candidate for cochlear gene therapy in humans. However, the surgical procedure remains challenging. Lee et al. recently showed that AAV9-PHP.B-eGFP injection into the neonatal mouse utricle results in the transduction of almost 100% of both IHCs and OHCs in the cochlea and the vestibular end organs with no deleterious effects on hearing and balance [32]. This vector and the utricle injection approach are, therefore, more suitable for the delivery of transgenes into the cochlea and vestibular organs of mice with hearing and balance defects. 

Another synthetic vector, AAV2.7m8, was generated for retinal gene therapy via an in vivo-directed evolution approach involving the screening of AAV libraries with diverse capsid protein modifications for the efficient transduction of mouse retinal photoreceptors [80]. Isgrig et al. recently showed that AAV2.7m8 is a powerful vector that transduces both IHCs and OHCs highly efficiently in all cochlear turns (mean transduction rate of 83%) [76]. AAV2.7m8 transduces vestibular hair cells less efficiently (28%) and OHCs more efficiently than Anc80L65 [25]. Moreover, AAV2.7m8 also mediates the highly efficient transduction of leucine-rich repeat-containing G-protein coupled receptor 5 (LGR5)-positive supporting cells, which have progenitor cell-like properties promoting hair cell regeneration [81,82]. The AAV2.7m8 vector could, therefore, considerably expand the potential applications for cochlear gene therapy.

Finally, a hybrid gene delivery system, exosome-associated AAV (exo-AAV), has been developed to increase transduction efficiency for both IHCs and OHCs. Exosomes are lipid-based extracellular vesicles involved in intercellular communication that can potentially be used to carry therapeutic nucleic acids and proteins [83,84,85]. The delivery of exo-AAV1-GFP into neonatal mouse cochlea by RWM or cochleostomy injection significantly increased the efficiency of cochlear hair cell transduction: more than 95% of IHCs and about 50% of OHCs were transduced after both RWM injection and cochleostomy. In the vestibular end organs, GFP-positive hair cells were evident in the utricle and ampullas of the lateral semicircular canals following both RWM injection and cochleostomy. In the utricle, there were 2.3 times more transduced GFP-positive hair cells than untransduced hair cells. The intracochlear injection of exo-AAV1-Lhfpl5-GFP in *Lhfpl5*^−/−^ mice improved hearing thresholds at frequencies from 4 to 22 kHz, and rescued the balance dysfunction in these mice (improving both circling and head tossing behaviors) [36]. The AAV vectors for in vivo gene delivery in animals described in publications over the last 10 years are summarized in Table 1, Table S3.

Improvements in the control of spatiotemporal expression and the amount of therapeutic agent required are anticipated in the next few years, through studies identifying the most efficient promoters and routes of administration to the inner ear. The choice of vector type depends on the therapeutic goal, the target cells and the gene to be transferred, but remains limited by our knowledge of the various vectors available.

#### 4.2.2. Non-Viral Delivery

Non-viral delivery offers a powerful complementary approach for delivering therapeutic agents to the inner ear. Non-viral delivery is less efficient than viral systems but more flexible and safer. Cationic liposomes, polymeric nanoparticles, biolistic materials, and electroporation are used for non-viral delivery. These vectors are easy to use, potentially safe for use in humans (without the adverse effects of virus integration in human DNA), and are less toxic and immunogenic than viral vectors [92]. Cationic liposome-mediated gene transfer has been widely used in vitro and in vivo for cancer gene therapy research [93]. Cationic liposomes form a bi-layered phospholipid structure that protects nucleic acids from degradation and antibody neutralization. They fuse to the cellular membrane, due to their cationic charge, facilitating the delivery of nucleic acids to the cytosol. Several in vitro and in vivo studies of the use of cationic liposomes to mediate transgene expression in the inner ear have been performed (Table 2). For example, Lipofectamine 2000 is a cationic liposome, which, despite its potential cytotoxicity, has been used for cochlear gene delivery in vitro and in vivo. Neonatal organs of Corti have been efficiently transfected with plasmids containing Math1, with Lipofectamine [94]. Several studies have tested other liposome formulations for the delivery of GFP or beta-galactosidase reporter genes into the cochlea via RWM administration [95,96,97]. The GFP or beta-galactosidase was expressed for up to 14 days in various cell types, including spiral limbus cells, spiral ligament cells, and spiral ganglion neurons. The delivery of cre-recombinase and Cas9-single guide-RNA nuclease complexes into the neonatal mouse inner ear in vivo resulted in 90% recombination and 20% Cas9-mediated genome modification in OHCs [98]. In a recent study, Gao et al. injected Cas9–guide RNA–lipid complexes targeting the *Tmc1*^Bth^ allele into the cochlea of neonatal Beethoven mice (model of DFNA36 dominant deafness); this treatment considerably reduced progressive hearing loss and improved sensory hair cell survival [99].

Drugs and genes have been successfully encapsulated in nanoparticles to prolong the time for which therapeutic agents remain in the bloodstream and to protect them against enzymatic destruction. Several synthetic or naturally polymeric nanoparticles have been developed and tested for gene delivery. They include dendrimers, polyethylenimine, dextran, chitosan, and poly (lactic-co-glycolic acid (PLGA) [100]. Most of these polymers readily associate with DNA molecules to form a polymer-DNA-complex (polyplex). These cationic polyplexes then interact with the negatively charged cell surface, facilitating uptake into the cell by endocytosis and the release of DNA into the nucleus for gene expression. Rhodamine-encapsulated polylactic/glycolic acid (PLGA) nanoparticles placed on the RWM of guinea pig cochleae were subsequently detected in the scala tympani, still in nanoparticle form, indicating that PLGA nanoparticles can cross the RWM [101]. Polybrene-mediated integrin antisense oligonucleotide transfer in rat otocysts and otocapsule tissues has been used to study the effects of integrins on the growth and proliferation of cochlear epithelial cell lines [102]. Polyethylenimine (PEI) is another polymer used for cochlear gene transfer in guinea pigs, via cochleostomy and osmotic pump infusion. PEI-infused cochleas have an intact cellular and tissue architecture, with no signs of inflammation. Transfection was restricted to the perilymphatic fluid spaces, and no transfected cells were observed in the organ of Corti [103]. In cochlear organotypic cultures, hyperbranched polylysine nanoparticles (HPNPs) have been shown to transfect spiral ganglion neurons efficiently, although a few hair cells were also transfected. In vivo, the application of a gelatin sponge immersed in HPNPs to the rat RWM favored the distribution of HPNPs in the sensory hair cells, supporting cells, stria vascularis marginal cells, spiral ligament fibrocytes, and spiral ganglion neurons [104]. These polymers are potentially useful for the delivery of drugs to the cochlea via local application, due to their biocompatibility, biodegradability, and ability to maintain therapeutic drug concentration over extended time periods. However, their low transfection efficiency, lack of target cell selectivity, and potential cytotoxicity limit their application in clinical trials.

Biolistics is a mechanical method of non-viral gene delivery, also known as particle bombardment or the gene gun. This method is ideal for gene transfer to the skin, mucosa, or surgically exposed tissues within a confined area. DNA is deposited on the surface of gold particles, which are then “fired” (accelerated by high-pressure helium discharge or high-voltage electronic discharge) into cells or tissue. The gene gun-mediated transfection of mouse vestibular and cochlear sensory epithelia explants with Myo15a-EGFP-C2 resulted in a selective accumulation of the GFP-tagged myosin XVa at the tips of stereocilia 24 h after transfection, confirming the localization of native myosin XVa [105]. Moreover, the gene gun-mediated transfection of hair cells from *Myo15a*^sh2^ or *Whrn*^wi^ mutant mice with GFP-Myo15a or GFP-Whrn restored the characteristic staircase shape of the hair bundles of both cochlear and vestibular GFP-positive hair cells [106]. In both studies, hair cells were efficiently targeted, but few cells were transfected. Other disadvantages of the biolistic method are the transient nature of transgene expression and the cellular damage observed at the discharge site.

Finally, electroporation can be used for both in vitro and in vivo inner ear gene delivery. In this technique, high-voltage electric field pulses, generated by an electrode inserted into the cochlea, create transient pores in lipid membranes, through which charged extracellular DNA is taken up. This method directly delivers large transgenes without the need for a packaging cell line. Its major disadvantages are the low efficiency and specificity of transfection, and its potential toxicity. Several studies have used in vivo electroporation to induce transgene expression in inner ear cells, particularly for the ectopic expression of transcription factors. Ectopic expression of the transcription factor Math1 (encoded by the Atoh1 or Math1 gene) in nonsensory regions of cochlear explant cultures is sufficient to induce the formation of sensory clusters containing both hair cells and supporting cells [107,108]. The in-utero transfer of an Atoh1-GFP gene leads to the production of functional supernumerary hair cells in the mouse cochlea. The induced hair cells have bundles of stereocilia, attract neuronal processes, and express the ribbon synapse marker carboxy-terminal binding protein 2 [109]. In utero electroporation of Cx30-eGFP plasmid in *Cx30*^−/−^ mice resulted in efficient expression of CX30 in spiral limbus, stria vascularis, spiral ligament, organ of Corti, and spiral ganglion neurons. Cx30 transfection restored ABR thresholds and endocochlear potential in the P30 *Cx30*^−/−^ mice [110]. Electroporation has also been shown to be an efficient method for the ectopic expression of other transcription factors, such as Sox2, Neurog1, and NeuroD1, in nonsensory regions of cochlear explant cultures, thereby inducing the formation of neuronal cells [111]. Interestingly, cochlear implant electrodes have been used for close-field electroporation to deliver brain-derived neurotrophic factor (BDNF) and GFP to the cochlear perilymphatic canals of guinea pigs and to stimulate the survival and sprouting of spiral ganglion neurons [112]. 

The non-viral vectors used in gene therapy for genetic hearing loss studies are summarized in Table 2.

### 4.3. Gene- and Mutation-Specific Therapies

#### 4.3.1. Gene Replacement

The most common form of gene therapy involves the delivery of a functional or therapeutic “transgene” that replaces or complements the defective gene responsible for the disease. The transgene is delivered directly to target cells or is reconstituted in the target cell nuclei. Biallelic recessive mutations and loss-of-function dominant mutations can generally be treated by this strategy. Clinical trials of gene replacement therapy for the treatment of certain forms of blindness in humans, including Leber’s congenital amaurosis (*RPE65*, NCT00999609) [113,114,115] and choroideremia (*REP1,* NCT02407678) [116], as well as development of therapeutic approaches for inner ear and central hearing disorders [17,19,117] have raised hopes that such a strategy could also be applied to the treatment of auditory and/or vestibular conditions.

The only real way to cure hereditary/genetic hearing loss is to tackle its cause. Hearing loss is an extremely heterogeneous disorder, with up to 1000 different genes potentially involved [11] that either cause deafness directly (Mendelian) or enhance the effects of environmental or personal risk factors. About 140 deafness genes have been identified to date, but many others remain to be discovered (see http://hereditaryhearingloss.org/). Improvements in our understanding of the molecules involved in audition, and of the underlying molecular mechanisms in auditory sensory cells and their associated neurons have opened up new opportunities for developing targeted gene therapy [1,18,19].

Furthermore, gene therapy approaches used in other systems can greatly influence progress in the design and implementation of gene therapy for genetic hearing loss. The preliminary results of ongoing gene therapy clinical trials for non-syndromic hearing loss are pending, and several gene therapy trials for syndromic hearing loss are currently underway, including those for the autosomal recessive gene *MYO7A*, which causes deaf-blindness in Usher syndrome [118].

The groundwork for the use of this approach in the ear was laid by Lawrence Lustig’s team in a mouse model of congenital deafness linked to a defect of the VGLUT3 protein, a glutamate transporter present in the inner hair cells (IHCs) [20]. This team demonstrated that the transfer of serotype 1 AAV vectors carrying the functional Vglut3 gene, through the round window, restored hearing to almost normal levels one to two weeks after injection. The success of this treatment was due, in part, to the very high transfection efficiency reached, with almost 100% of the IHCs transduced. Interestingly, almost 90% of the mice receiving the injection on the day after their birth retained the ability to hear for 28 weeks. By contrast, this proportion fell to 10% for animals receiving injections on postnatal day 10 (P10). These findings demonstrate the utility of early gene injection to ensure effective and sustained hearing restoration, while highlighting the importance of finding the appropriate therapeutic window for each type of deafness.

Other studies on diverse models of deafness have since confirmed the efficacy of gene supplementation for correcting inner ear defects [22,23,25,88] (see Table 1). An almost total restoration of vestibular function and a less complete restoration of hearing were observed in models with defects of three molecules implicated in Usher syndrome (the leading cause of blindness and deafness in humans): harmonin, sans, and whirlin. Defects of these proteins underlie USH1C [25], USH1G [23], and USH2D [119], respectively; whirlin is also responsible for DFNB31 non-syndromic autosomal recessive hearing loss [88,120,121]. These proteins are involved in establishing the hair bundle structure and are components of the mechanoelectrical transduction machinery responsible for converting sound into changes in membrane potential [1,122]. In treated mice, the hair bundles of cochlear OHCs and IHCs and vestibular hair cells recovered a normal morphology after treatment, with an almost complete and long-lasting restoration of vestibular function. Measurements of otoacoustic emissions and of auditory evoked potentials demonstrated a partial recovery of hearing in these mice, particularly at low frequencies. In particular, the new synthetic AAV, Anc80L65, used to transfer the functional harmonin gene into the ear [25], enhanced harmonin gene expression in both the IHCs, and the more difficult to reach OHCs, constituting a major advance towards hearing restoration in cases of auditory problems of genetic origin involving defects of both IHCs and OHCs.

#### 4.3.2. Gene Suppression—RNA-Based Therapies

Antisense oligonucleotides (ASOs) and short interfering RNAs (siRNAs) or microRNAs (miRNAs) can be used to treat cases of dominant-negative deafness. ASOs are single-stranded, modified synthetic RNA or DNA sequences that selectively bind, via complementary base-pairing, to the RNA generated from the gene of interest; they alter mRNA processing or degrade the target transcript [123]. By contrast, siRNAs are small double-stranded RNA molecules that trigger the RNAi pathway. This pathway is a natural cellular defense mechanism against RNA viruses, which identifies pathogenic double-stranded RNAs and targets them for cleavage [124].

In USH1C, a form of Usher syndrome type 1, a frequent mutation of the harmonin gene has been reported in an American population of Arcadian origin. This mutation affects a cryptic splice site and leads to the production of truncated harmonin protein. This *USH1C* c.216G>A mutation was reproduced in mice, in which it caused early-onset, profound deafness, similar to that observed in USH1C patients [125]. Forty-seven different individual ASOs were screened and shown to bind the mutated DNA strand, blocking the defective cryptic splice site, and thereby promoting the correct splicing and production of normal harmonin protein. The ASO sequence providing the best results for splicing correction in vitro was then introduced into the mouse model in vivo. Surprisingly, a single intraperitoneal injection of the ASO restored vestibular and auditory function to near-normal levels in the treated mice (particularly for low frequencies, 8 to 16 kHz in mice). High levels of wild-type harmonin and a restoration of hair bundle morphology were observed [125]. Many unanswered questions remain concerning the mechanism of action of ASOs and their mode of transfer to the inner ear, particularly to the hair cells, following systemic injections. However, the use of antisense oligonucleotide sequences could be generalized to other types of mutations in causal genes for deafness.

Another approach based on RNA interference has been used in a model of dominant non-syndromic deafness, DFNA3, in which the connexin-26 gene is defective. Deafness forms with autosomal dominant mode of transmission (about 15% of cases) are generally progressive, and the pathogenic mechanism is often based on negative dominance (the abnormal protein produced from the mutated allele disrupts the function of the normal protein produced from the healthy allele). In such situations, therapeutic approaches usually involve inhibiting or deactivating the expression of the mutant allele without affecting the expression of the healthy allele. One way to achieve such selective inhibition of the mutant allele is to use RNAi. In this model, the placement of a resorbable gelatin sponge imbibed with liposomes enclosing the siRNA specific for the mutated connexin-26 against the RWM decreased mutant allele expression by 70%, and prevented the progression of hearing loss, without significantly decreasing expression of the wild-type allele [126]. Shibata et al. recently showed that a single neonatal intracochlear injection of an artificial miRNA in the Beethoven mouse, a murine model of DFNA36 non-syndromic human deafness caused by a dominant gain-of-function mutation in *Tmc1* (transmembrane channel-like 1), slowed the progression of hearing loss for up to 35 weeks [127]. Following this study, Yoshimura et al. tested this artificial miRNA in the adult Beethoven mouse. They performed intracochlear injections of an AAV vector carrying the appropriate sequence at P15 and P30, and showed that miRNA-mediated gene silencing slowed the progression of hearing loss, improved inner hair cell survival, and prevented the degeneration of bundles of stereocilia in the adult mice [128]. However, no restoration of hearing or morphology was observed in mice treated at P84-P90, demonstrating that the effect of treatment depends on the age of the animal treated.

Together, these findings demonstrate that RNA-based approaches can be used in vivo, with satisfactory levels of specificity and efficacy, to prevent hearing loss due to a dominant negative effect in genetic forms of deafness. They represent a significant step towards the translation of this approach to human subjects.

#### 4.3.3. CRISPR/Cas9-Based Genome Editing

Gene editing strategies differ according to the form of deafness treated and its mode of transmission. For certain recurrent mutations, it is possible to correct a specific region of the defective gene through diverse targeted molecular approaches. Genome editing tools with higher levels of performance have progressively been developed, based on the use of ZFNs (zinc finger nucleases) [129], TALENs (transcription activator-like effector nucleases) [130], and CRISPR/Cas9 (clustered regularly interspaced short palindromic repeats and CRISPR-associated protein 9), which can be used to cut and modify DNA at precise sites [131]. Due to their ease of use and efficacy, these tools (particularly CRISPR/Cas9 approaches) are now being used to correct one or several sites in the genome simultaneously, with the aim of providing durable treatments restoring normal function in patients [132]. In auditory research, this approach is effective for dominant forms of genetic deafness, in which most of the mutations are single-nucleotide substitutions. Gao et al. were the first to develop a genome-editing approach to target DFNA36, a non-syndromic dominantly inherited form of deafness [99]. Cationic lipid-mediated Cas9–single guide RNA complex delivery was targeted to the mutant *Tmc1* allele in hair cells of P1 Beethoven mice, to slow the progression of hearing loss. In the mice receiving the injections, a slight restoration, limited to frequencies between 8 and 23 kHz, was observed eight weeks post-treatment. This modest degree of hearing preservation (less than 20 dB) is consistent with the small number of hair cells corrected, and, perhaps, a lack of specificity of Cas9 for the mutant allele, which differs from wild-type *Tmc1* by only a single base pair. Efforts were recently made to reduce this nonspecificity, by using a proto-spacer-adjacent motif (PAM) variant of *Staphylococcus aureus* Cas9 (SaCas9-KKH) to target the mutant allele more selectively and efficiently, without targeting the wild-type allele, in Beethoven mice [133]. Intracochlear neonatal AAV-mediated SaCas9-KKH-gRNA delivery has been shown to prevent deafness in *Tmc1*^Bth/WT^ mice for up to one year post-injection, and to preserve normal hair bundle morphology in both IHCs and OHCs.

These recent studies highlight the wide range of therapeutic approaches available, which can be adapted for a particular gene and therapeutic goal. These diverse approaches are increasingly used and are revolutionizing personalized medicine and gene therapy.

### 4.4. “Gene-Independent” Approaches—A Common Strategy for Several Forms of Deafness 

As mentioned above, several hundred different genes are likely to be involved in the various forms of deafness. The idea of correcting each of these defects is attractive but would be difficult to achieve in the short-to-medium term. Gene therapies applicable to several forms of deafness at once, whether hereditary or acquired, would be a promising approach for preserving or recovering a functioning auditory structure.

Since the discovery of stem cells and their capacity to repair damaged tissues in regenerative therapy approaches, considerable progress has been made towards the development of protocols for guiding the controlled differentiation of stem cells into the various cell types of the inner ear. The greatest potential of these cell lines in the domains of balance and hearing lies in their use for establishing cellular models of diverse forms of hearing loss, particularly in three-dimensional cultures. Moreover, many studies focus on the identification of molecules able to protect or repair the activity of auditory sensory cells and/or neurons in pharmacological therapy approaches (for review see [134]).

#### 4.4.1. Auditory Hair Cell Regeneration

Efforts have been made to promote the regeneration or replacement of defective hair cells as a means of protecting hearing or preventing its degradation. Encouraging results have been obtained in vitro and in animals, particularly by overexpressing the Math1 transcription factor, which is both necessary and sufficient to induce the differentiation of hair cells in the mammalian cochlea. In guinea pigs rendered deaf by exposure to ototoxic drugs, leading to the destruction of the native hair cells, the authors of one study showed that the transfer of an adenovirus carrying the Math1 gene triggered the formation of new auditory hair cells through the trans differentiation of support cells [135]. Similar results were obtained in mice and rats, in which a partial restoration of hearing thresholds was observed. The first trial of gene therapy for the treatment of deafness in humans, based on this approach, was recently launched in the US by the team of Professor Hinrich Staecker (NCT02132130). This trial includes volunteers aged between 18 and 70 years, with almost total hearing loss, but with a large number of supporting cells remaining in the cochlea. The objective is to deliver a Math1-expressing adenovirus to the cochlea of these patients, with the aim of evaluating the safety of the treatment. The results of this trial are expected shortly. Information on its progression and outcomes will certainly benefit actions for future inner ear clinical trials. Indeed, a study in mice has shown that the injection of Math1 into two-week-old mice, a stage equivalent to puberty in humans, has little or no effect. This approach is not currently suitable for application to deafness forms of genetic origin unless designed to correct the genetic mutation responsible for the defect. 

#### 4.4.2. Protective Local Treatments

Another promising avenue explores the survival pathways of hair cells and their associated neurons. Indeed, whether the cause of deafness is a genetic abnormality, noise, or ototoxic agents, an active cell death program is often observed in these sensory cells. Several studies in recent years have investigated the transfer of genes encoding growth factors or mitotic agents to block the degeneration of inner ear neurons. These approaches aim to keep as many neurons alive as possible, to guarantee a high performance from cochlear implants. The beneficial effects of molecules such as neurotrophin-3 (NT-3) and BDNF for protection against cochlear neuron degeneration have recently been demonstrated in various animal models [3]. The local delivery of NT-3 to the round window niche of noise-exposed mice can rescue cochlear synaptopathy, through the regeneration of cochlear synapses and the preservation of cochlear nerve fibers [136]. In one recent study, gene therapy was combined with a cochlear implant in a guinea pig model [112]. In this study, the growth factor BDNF was delivered via a plasmid containing both the BDNF and GFP genes under the control of the CMV promoter. GFP expression was used as an indicator for in situ identification of the cells expressing BDNF. This study involved considerable technical prowess. The entry of the vector into cells was induced in situ by a 20 V electrical discharge from the hearing implant, opening the pores of the cells to facilitate the integration of plasmids from the surrounding medium. Treatment in guinea pigs rendered deaf by ototoxic drugs led to a regrowth of the nerve fibers after two weeks, theoretically making the use of their cochlear implants more effective. No auditory tests were used to compare the auditory performances of treated and untreated animals with cochlear implants, but this study illustrates the enormous potential of approaches combining the use of cochlear implants with tissue engineering [3,112,137]. Such approaches aim to keep as many neurons as possible alive, or to favor neuronal regrowth, with the goal of improving the electrode interface of the cochlear implants, resulting in better neuronal transmission and auditory performance. 

More detailed studies of these therapeutic approaches in animal models are required before their application in humans can be envisaged. However, mixed treatments combining at least two of the therapies listed above may be the most appropriate way to ensure the protection of auditory activity.

### 4.5. From Animals to “One Day” in Humans: the Promises and Challenges of Preclinical Inner Ear Gene Therapy Trials

Therapeutic studies in animals are progressing very rapidly; they are expected to help bridge the gap between proof-of-concept preclinical studies and clinical trials in patients, thus supporting translational medicine with the launch of clinical trials. However, it is still too soon to predict the success of such trials and the application of these treatments to patients. For a particular cochlear gene therapy to be considered successful, it must at least match the efficacy and safety achieved with the existing cochlear implants. Studies using a combination of different approaches acting on multiple parameters are already underway, to address a number of important unanswered questions: 

1- How can we regenerate auditory hair cells? Math1 acts as the cornerstone of this therapeutic strategy, but the longevity of the pathway and efficacy in animal models are currently unknown. The results of clinical tests currently underway will provide some answers to these questions. However, other avenues could undoubtedly be explored, such as the signals involved in inner ear regeneration in non-mammalian vertebrates. Non-mammalian vertebrates can spontaneously replace or regenerate hair cells and innervation lost following acoustic trauma or damage caused by ototoxic agents. Nevertheless, once the conditions for such regeneration have been identified, we will need to determine how they can be reactivated in mammalian ears and how to test their maintenance over time in an environment that has become non-permissive.

2- How can we reproduce or maintain cochlear tonotopy? As described above, the mature cochlea displays tonotopic functioning established during the morphogenesis and differentiation of the auditory organ. The signals controlling the variation from the base to the apex of the auditory organ are mostly absent at adult stages. The recovery of normal hearing after hearing loss requires the repaired or newly differentiated cells to have the appropriate properties for their position along the length of the cochlea, and the establishment of appropriate mechanical coupling with the surrounding support cells and innervating neurons. In future models of hearing restoration, it will be interesting to document the correlations between the functional recovery observed and the molecular and cellular properties of hair cells and their associated neurons, along the length of the cochlea. Such data would shed light on the number of sensory cells required, and the degree of cochlear tonotopy required to achieve a gain of given X dB level or almost “normal” hearing.

3- Which treatment for which form of deafness? Ideally, the development of appropriate personalized treatments requires identification of the pathophysiological mechanism of the mutation or deafness form, definition of the natural course of the disease, and the treatment window. Such knowledge would help to determine the respective contributions of preventive and corrective therapies to the treatment of forms of deafness differing in terms of age at onset, severity, progression, and target cell types. 

4- Which vectors, for which cell type, and which administration route? The choice of vector is a key factor determining the success of gene therapy, and access to the inner ear remains a challenge that must be overcome in the development of new therapies (gene, cell, and/or pharmacological). For gene therapy, the identification of appropriate vectors (natural and/or synthetic viruses), targeting the cells of interest and ensuring the durable and certain expression of the target gene, is a priority. Despite the potential benefits of viral gene therapy, the use of viral vectors in the clinical setup is limited by the possibility of tumorigenesis and putative adverse effects from virus integration into human DNA. Other transfer vectors, such as liposomes, metallic nanoparticles, polymers, and gelatins, are promising, due to their high loading capacity (for genes or drugs) and their effective absorption in the inner ear. Further studies are required, to increase the efficacy and definition of target cells according to the mode of administration (diffusion or injection). Indeed, although the hair cells seem to be the principal cells affected in about 50% of all cases of hearing loss, other cell types (e.g., support cells (fibrocytes, epithelial cells, mesenchyme cells) and the neurons of the inner ear or auditory cortex) may also be defective. 

## 5. Conclusions

Continuous technical progress in the field of hearing aids and cochlear implants will ensure that these devices continue to play a major role in the treatment of hearing loss, but the search for additional inner ear tissue- and cell-based treatment solutions merit further consideration. Deafness encompasses hundreds of extremely diverse genetic and phenotypic forms of hearing loss. The diverse causes of deafness, the diversity of the target cells, and the various pathogenic processes and disease etiologies require the development of diverse, adapted treatment solutions. The identification of precise and accurate clinical endpoints in patients, as a function of the causal gene, is required, to improve the evaluation of treatment efficiency in humans, and to develop appropriate personalized therapy. As shown by recent progress in animal models, gene therapy is a promising approach to preventing or slowing the loss of hearing and/or balance. Gene therapy is not limited to the addition of a healthy copy of the defective gene, but may also involve gene silencing or editing via nucleic acid-based strategies involving antisense oligonucleotides, siRNA, microRNA, or nuclease-based gene editing. Due to the complexity of implementation and the potential risks associated with the inappropriate use of these approaches, it is too early to predict the likely contribution of any particular approach to the restoration of hearing in patients with hearing impairments. Rapid advances and breakthroughs are still needed to expand gene therapy applications for the treatment of hearing loss in clinical practice. These advances include: (i) The identification of safe and effective delivery routes for genes and proteins, suitable for use in clinical practice, including viral and non-viral approaches, (ii) The identification of new vectors for the more efficient and specific targeting of inner ear cells, including dual vector systems for larger transgenes; (iii) Enhanced gene editing, with improved nuclease variants (new Cas9 enzymes, base, and prime editors), with or without regulatory proteins/microRNAs to improve repair mechanisms); and (iv) The establishment, in a living host, regardless of the treatment used, of the optimal therapeutic window, the long-term therapeutic effects, and interactions with other organs, to monitor the semi-immune-privilege of the inner ear. There are currently more than 3000 clinical trials underway involving gene therapy. Lessons from these gene therapy approaches in other systems could greatly improve the design and implementation of gene therapy for cochlear and vestibular disorders. During his inaugural speech at the Faculty of Sciences (Lille, 7 Dec 1854), Louis Pasteur said “Chance only favors the prepared mind”. It is therefore essential that “together”, scientists, clinicians, audiologists and all actors in the inner ear field, become better prepared, ready to transform today’s promises into tomorrow’s successful therapies.

## Figures and Tables

**Table 1:** A summary of adeno-associated virus (AAV) vectors used in gene therapy for genetic hearing impairments and vestibular dysfunction.

Gene Name; Deafness Locus	AAV Vector	Animal Model	Route of Delivery; Age at Delivery	Target Cells/Outcomes	References
*Vglut3*; DFNA25	AAV1-mVglut3	*Vglut3*^−/−^ mice	RWM; P1-P3, P10-P12	Transduction of almost 100% IHCs, normal ABR thresholds and waveforms at both low and high frequencies, up to 28 weeks post injection. Earlier delivery increases hearing recovery longevity.	[20]
*Gjb2 (Cx26)*; DFNB1	AAv2/1-CB7-Gjb2 AAV2/1-CB7-Gjb2-GFP	*Cx26*^fl/fl^*Foxg1-Cre^+/^*^−^ mice	Cochleostomy; P0-P1	High transduction efficiency of supporting cells, especially outer sulcus cells. Very low transduction of sensory hair cells, but improved hair cells and spiral ganglion neurons survival. No restoration of hearing sensitivity.	[35]
	AAV2/5-CMV-Gjb2	*Cx26*^fl/fl^P0-Cre mice	RWM; P0, P42	Transduction of supporting cells, the spiral ligament fibrocytes and the spiral limbus. Preserved structure of OHCs, IHCs, and supporting cells, and improved ABR thresholds at neonatal stage, but not in adult mice.	[86]
*Tmc1*; DFNB7/11 Tmc1^Bth/+^; DFNA36	AAV2/1-CBA-Tmc1 AAV2/1-CBA-Tmc2	*Tmc1*^−/−^ and *Tmc1*^Bth^ mice	RWM; P0-P2	Restoration of IHCs function. Partial recovery of ABR thresholds in *Tmc1*^−/−^ mice injected either with either AAV2/1-CBA-Tmc1 or AAV2/1-CBA-Tmc2. No recovery of OHC function, due to low viral transduction rates in OHCs.	[22]
	AAV2/Anc80L65-CMV-Tmc1-WPRE, AAV2/Anc80L65-CMV-Tmc2-WPRE, AAV2/Anc80L65-CMV-Tmc1EX1-WPRE, AAV2/Anc80L65-CMV-EGFP-WPRE	Wild-type and *Tmc1*^−/−^ mice	RWM; P0-P2, P4, P7, P14, and P30	Cochlea: High transduction of both IHCs and OHCs at neonatal stage. Rescued sensory function in mature hair cells, and enhanced hair cell survival. Partial recovery of ABR thresholds, especially at the low frequencies. Vestibular end-organs: High transduction of vestibular hair cells, and enhanced viability of hair cells. Restoration of vestibular behavior and balance function even at mature stages.	[75]
*Whrn*; DFNB31; USH2D	AAV2/8-CMV-Whrn-GFP	Whirler (*Whrn*^wi/wi^) mice	RWM; P1-P5	Transduction of ~15% of IHCs, and any of OHCs. No improvement in hearing sensitivity. Restoration of stereocilia length and hair bundle morphology and increase in IHCs survival.	[87]
	AAV2/8-CMV-Whrn-GFP	Whirler (*Whrn*^wi/wi^) mice	PSCC; P4	Cochlea: Normal stereocilia bundles morphology. Successful transduction of IHCs, and partial restoration of hearing for at least 4 months. Vestibular end-organs: Efficient transduction of vestibular hair cell. Restoration of utricular hair cells morphology. Normal vestibular behavior and balance function for at least 4 months, and improved vestibular evoked potentials (VsEPs).	[88]
*Pjvk*; DFNB59	AAV2/8-Pjvk-IRES-eGFP AAV8-Pjvk-IRES-eGFP	*Pjvk*^−/−^ mice	RWM; P3	Partial restoration of ABR thresholds, normal ABR waveforms and wave amplitudes.	[21]
*MsrB3*; DFNB74	rAAV2/1-CMV-MsrB3-GFP	*MsrB3*^−/−^ mice	Otocysts; E12.5	High transduction efficiency of both IHCs and OHCs in all cochlear turns. Preserved hair cells, rescued morphology of stereociliary bundles, and normal ABR thresholds at both low and high frequencies at P28.	[89]
*Clrn1*; Ush3A	AAV2/2-CAG-Clrn1-UTRs AAV2/8-CAG-Clrn1-UTRs	*Clrn1*^−/−^ and KO-TgAC1 mice (Transgene Atoh1-enhacer-Clrn-1 UTRs)	RWM; P1-P3	Preserved hair bundle morphology at P100. Normal click-evoked ABR thresholds and waveforms at P100.	[90]
	AAV2/8-CAG-Clrn1-IRES-eGFP	*Clrn1*^ex4−/−^ and *Clrn1*^ex4fl/fl^ *Myo15*−*Cre*^+/−^ mice	RWM; P1-P3	High transduction of both IHCs and OHCs. An almost complete rescue of hearing for low and high frequencies in *Clrn1*^ex4fl/fl^*Myo15*-Cre^+/−^mice. Prevention of the synaptic defects and durably preservation of the stereocilia hair bundles morphology up to P12.	[26]
	AAV2/9.PHP.B-CBA-Clrn1-eGFP	*Clrn*^−/−^mice	RWM; P0-P1, P30	Cochlea: High transduction of both IHCs and OHCs at neonatal stage. Almost all IHCs from apex to base transduced, but no OHC transduction at adult stage. Robust hearing rescue at low frequencies. Vestibular end-organs: Robust transduction of vestibular hair cells	[27]
*Ush1c*; DFNB18	AAV2/Anc80L65-CMV-Harma1 AAV2/Anc80L65-CMV-Harmb1	*Ush1c* c.216G>A knockin mice (Acadian mutation)	RWM; P0-P1, P10-P12	Cochlea: High transduction efficiency of both IHCs and OHCs. Normal ABR thresholds in mice injected with harmonin-b1 alone or harmonin-a1/b1 together, particularly at low frequencies. Normal hair bundle morphology along the entire organ of Corti at 6 weeks of age. Vestibular end-organs: Restoration of balance behaviors	[25]
*Sans; USH1G*	AAV2/1-CAG-Sans-eGFP AAV2/2-CAG-Sans-eGFP AAV2/5-CAG-Sans-eGFP AAV2/8-CAG-Sans-eGFP	*Ush1g*^−/−^ mice	RWM; P2.5	Cochlea: AAV2/1 and AAV2/2 and AAV2/5 injections mostly transduced supporting cells of the organ of Corti. AAV2/8 injection transduced IHCs with greater efficiency at the apex of the cochlea than at the base, whereas OHCs were transduced with roughly the same efficiency at the base and at the apex. Restoration of hair bundle morphology, and improved hearing thresholds. Vestibular end-organs: AAV2/8 transduced the vast majority of vestibular hair cells, restored morphology of stereociliary bundles, and durably rescued balance defects.	[23]
*Lhfpl5*; DFNB66/67	Exo-AAV1-CBA-GFP Exo-AAV1-CBA-HA-Lhfpl5	Wild-type and *Lhfpl5*^−/−^ mice	RWM; Cochleostomy; P0-P1	Cochlea: Both RWM and cochleostomy injection transduced with high efficiently both IHCs and OHCs. Cochleostomy also transduced spiral ganglion neurons and supporting cells. Improved hearing thresholds at frequencies from 4 to 22 kHz. Vestibular end-organs: Robust transduction of vestibular hair cells via both RWM and cochleostomy injections. Restoration of balance behaviors.	[36]
*Otof*; DFNB9	Dual vector: AAv2/quadY-F-smCBA-Otof N term-AA1-816-SD and AAv2/quadY-F-smCBA-Otof C term-AA817-1992-SA-pA. ALK bridge 3′ to SD and 5′ to SA, respectively.	*Otof*^−/−^ mice	RWM; P10, P17, and P30	High transduction efficiency of IHCs. Durable restoration of otoferlin expression in transduced inner hair cells. Normal ABR thresholds for both click and tone-burst stimuli in treated mice. Restoration of ABR wave I latency, and partial recovery of ABR wave I amplitude.	[73]
	Dual 5′-AAv2/6-TS and 5′-AAV2/6-hybrid: hbA-CMVe-eGFP-P2A, 5′-*Otof* CDS-exon 1-21-SD. Dual-3′AAV2/6-TS and 3′-AAV2/6-hybrid: SA-3′*Otof* CDS-exon 22-46-WPRE-pA. ALK bridge 3′ to SD and 5′ to SA, respectively	*Otof*^−/−^ mice	RWM; P6-P7	Highly efficient transduction of IHCs, supporting cells, and spiral ganglion neurons. Full recovery of fast exocytosis in *Otof*^−/−^ IHCs. Normal click-evoked ABR thresholds and waveforms (particularly, waves II-V), and increased ABR wave amplitudes.	[74]
*Slc26a4*; DFNB4	rAAV2/1-CMV-Slc26a4-tGFP	*Slc26a4*^−/−^ and Slc26a4^tm1Dontuh/tm1Dontuh^ mice	Otocysts; endolymphatic sac; E12.5	Cochlea: Restoration of hearing function, but variable hearing phenotype between injected mice. Preservation of both OHCs and IHCs at 5 weeks of age. Vestibular end-organs: Transient pendrin expression prevented enlargement of the membranous labyrinth but failed to restore otoconia formation and the acquisition vestibular function.	[91]

RWM, round window membrane; PSCC, posterior semicircular canal; IHCs, inner hair cells; OHCs, outer hair cells: ABR, auditory-evoked brainstem response; CBA, chicken β-actin; SD, splice donor site; SA, acceptor site; TS, trans-splicing; CDS, coding sequences; hbA, human beta-actin promoter; CMVe, cytomegalovirus enhancer; pA, polyadenylation signal; WPRE, woodchuck hepatitis virus post-transcriptional regulatory element; ALK, Alkaline Phosphatase; tGFP, turbo green fluorescent protein.

**Table 2:** Non-viral vectors used for inner ear gene therapy.

Non-Viral Vector	Transgene	Animal Model	Route of Delivery	Targeted Cells/ Outcomes	References
**Cationic Liposomes**					
Liposomes	β−gal plasmid	Guinea pig	RWM or cochleostomy and osmotic minipump infusion	Spiral limbus, spiral ligament, Reissner’s membrane, and spiral ganglion neurons	[95]
Liposomes	eGFP plasmid	Mouse	Gelfoam on RWM	Auditory hair cells, spiral ganglion neurons, spiral ligament, and stria vascularis	[92,96,97]
Lipofectamine 2000	Math1 plasmid (pcDNA6.2/C-EGFP-Math1)	Rat	Organ of Corti-derived cell line	Transfection of fibrocytes, spiral ganglion neuron and hair cell-like cells. Very low transfection efficiency (2.9%)	[94]
Lipofectamine 2000, Lipofectamine RNAiMax	Cas9:sgRNA complexes fused to (−30) GFP-Cre	Atoh1-GFP mice	Cochleostomy	Up to 20% Cas9-mediated genome modification in outer hair cells (loss of GFP expression near the injection site after 10 days)	[98]
Lipofectamine 2000	Cas9:sgRNA complexes targeting the Tmc1^Bth^ allele	*Tmc1*^Bth/+^ mice	Cochleostomy	Higher hair cell survival rates, improvement in ABR thresholds for the frequencies between 8–23 kHz, and greater ABR waves amplitudes with almost normal waveform pattern.	[99]
**Polymeric nanoparticles**					
Poly (lactic-co-glycolic acid) nanoparticles (PLGA)	Rhodamine	Guinea pig	Gelfoam on RWM	Scala tympani	[101]
Polybrene	Integrin subunits antisense oligonucleotides	Rat	Organ of Corti-derived cell line	Efficient inhibition of integrin subunits expression.	[102]
Polyethylenimine	eGFP plasmid	Guinea pig	Cochleostomy and osmotic minipump infusion	GFP expression in fibrocytes lining the scala vestibuli and scala tympani, mesenchymal and epithelial cells of Reissner’ membrane, and fibrocytes of the spiral ligament. No transfection in the organ of Corti or stria vascularis.	[103]
Dendritic polymer (hyperbranched poly-L-Lysine nanoparticle; HPNP)	eGFP plasmid	Rat	Gelatin sponge on RWM	Efficient GFP expression in hair cells, supporting cells, the stria vascularis marginal cells, the spiral ligament fibrocytes, and spiral ganglion neurons	[104]
**Biolistic (Gene Gun)**					
Gold particles	pEGFP-MyoXVa	Mouse	Organ of Corti explants	MyoXVa-GFP expression at the tips of stereocilia	[105]
Gold particles	pEGFP-MyoXVa or pEGFP-Whrn	*Myo15a*^sh2^ or *Whrn*^wi^ mice	Organ of Corti explants	Restoration of hair bundle staircase shape in both cochlear and vestibular GFP-positive hair cells	[106]
**Electroporation**					
Electroporation	Math1-eGFP plasmids	Mouse	Organ of Corti explants	GFP expression in greater epithelial ridge and stereociliary bundles in the hair cells	[107,108]
Electroporation	Atoh1 (Math1)-GFP plasmid	Mouse	*In utero* (microinjected into the E11.5 otic vesicle)	Efficient GFP expression in hair cells and supporting cells.	[109]
Electroporation	pCMV-Cx30-eGFP plasmid	*Cx30*^−/−^ mice	*In utero* (microinjected into the E11.5 otic vesicle)	Efficient CX30 expression in spiral limbus, organ of Corti, stria vascularis, spiral ligament, and spiral ganglion neurons at P30. Normal ABR thresholds and endocochlear potential at P30	[110]
Electroporation	Transcription factors: Sox2, Neurog1, and Neurod1	Mouse	Organ of Corti explants	Ectopic expression of these transcription factors in nonsensory regions of cochlear explant cultures induce the formation of neuronal cells	[111]
“Close-field” electroporation through cochlear implant electrodes	BDNF-GFP	Guinea pig deafened by kanamycin-furosemide treatment	RWM cochlear implant	Stimulated survival and regeneration of spiral ganglion neurons.	[112]

RWM, round window membrane; ABR, auditory-evoked brainstem response; eGFP, enhanced green fluorescent protein.

## Supplementary Materials

The following are available online at https://www.mdpi.com/2077-0383/9/7/2309/s1, Table S1: Non-syndromic hearing loss loci and genes in human. Table S2: Comparison of different routes of delivery to the inner ear. Table S3: Adeno-associated virus (AAV) vectors used in inner ear gene therapy studies.
