# Supplementary material for: Inner Ear Gene Therapies Take Off: Current Promises and Future Challenges
Source: J Clin Med. 2020 Jul 21;9(7):2309. doi: 10.3390/jcm9072309 (PMC7408650; doi:10.3390/jcm9072309)
Supplement: Supplementary file 1 [file jcm-09-02309-s001.pdf]

Table S1. Loci and genes underlying non-syndromic hearing loss that are grouped into several functional categories.

**Non-syndromic hearing loss human genes in the auditory hair cells**		
**Hair bundle development and functioning**		
**Locus**	**Gene**	**OMIM**
DFNB6	*TMIE* (Transmembrane inner ear-expressed gene)	607237
DFNB7/11, DFNA36	*TMC1* (Transmembrane channel-like protein 1)	606706
DFNB15	*GIPC3* (GIPC PDZ domain-containing family, member 3)	608792
DFNB28	*TRIOBP* (TRIO and F-actin-binding protein)	609761
DFNB16	*STRC* (stereocilin)	606440
DFNB24	*RDX* (radixin)	179410
DFNB25	*GRXCR1* (glutaredoxin and cysteine rich domain containing 1)	613283
DFNB32	*CDC14A* (cell division cycle 14A)	603504
DFNB36	*ESPN* (espin)	606351
DFNB44	*ACY1* (adenylate cyclase 1)	103072
DFNB66	*DCDC2* (doublecortin domain containing 2)	605755
DFNB63	*LRTOMT* (leucine rich transmembrane and O-methyltransferase domain containing)	612414
DFNB67	*LHFPL5* (LHFPL tetraspan subfamily member 5)	609427
DFNB79	*TPRN* (taperin)	613354
DFNB82	*GPSM2* (G protein signaling modulator 2)	609245
DFNB88	*ELMOD3* (ELMO domain containing 3)	615427
DFNB2/, DFNA11	*MYO7A, USH1B* (myosin VIIA)	276903
DFNB23	*USH1F, PCDH15* (protocadherin related 15)	605514
DFNB12	*USH1D, CDH23* (cadherin 23)	605516
DFNB18	*USH1C/ USH1C* (USH1 protein network component harmonin)	605242
DFNB31	*USH2D/ WRHN* (whirlin)	607928
DFNB48	*CIB2* (calcium and integrin binding family member 2)	605564
DFNB57	*PDZD7* (PDZ domain containing 7)	612971
DFNAi	*MYO1C* (myosin IE)	601479
DFNB30	*MYO3A* (myosin IIIA)	606808
DFNA22	*MYO6* (myosin VI,)	600970
DFNB3	*MYO15A* (myosin XVA)	602666
DFNB99	*TMEM132E* (transmembrane protein 132E)	616178
DFNB100	*PPIP5K2* (diphosphoinositol pentakisphosphate kinase 2)	611648
DFNB101	*GRXCR2* (glutaredoxin and cysteine rich domain containing 2)	615762
DFNB102	*EPS8* (epidermal growth factor receptor pathway substrate 8)	600206
DFNB103	*CLIC5* (chloride intracellular channel 5)	607293
DFNB103B	*FAM65B* (RHO family interacting cell polarization regulator 2,)	611410
DFNB106	*EPS8L2* (EPS8 like 2)	614988
DIAPH3	*AUNA1* (diaphanous related formin 3)	614567
DFNA1	*DIAPH1* (diaphanous related formin 1)	602121
DFNA20/26	*ACTG1* (actin gamma 1)	102560
DFNA27	*REST* (RE1 silencing transcription factor)	600571
DFNA50	*MIR96* (microRNA 96)	611606
DFNA67	*OSBPL2* (oxysterol binding protein like 2)	606731
DFNA76	*PLS1* (plastin 1)	602734
DFNB77	*LOXHD1* (lipoxygenase homology domains 1)	613072
DFNB84A/DFNA73	*PTPRQ* (protein tyrosine phosphatase receptor type Q)	603317
**Non-syndromic hearing loss human genes in the auditory hair cells**		
**Synaptic transmission**		
DFNB9	*OTOF* (otoferlin)	603681
DFNB15	*GIPC3* (GIPC PDZ domain containing family member 3)	608792
DFNB18	*USH1C* (USH1 protein network component harmonin)	605242
DFNB86, DFNA65	*TBC1D24* (TBC1 domain family member 24)	613577

DFNB93	*CABP2* (calcium binding protein 2)	607314
DFNB94	*NARS2* (asparaginyl-tRNA synthetase 2)	612803
DFNB102	*EPS8* (epidermal growth factor receptor pathway substrate 8)	600206
DFNB107	*WBP2* WW domain binding protein 2 ()	606962
DFNB108	*ROR1* (receptor tyrosine kinase like orphan receptor 1)	602336
DFNB114	*GRAP* (GRB2 related adaptor protein)	604330
D FNA25	*S LC17A8* (solute carrier family 17 member 8)	607557
DFNA68	*HOMER2* (homer scaffold protein 2)	604799
DFNA71	*DMXL2* (Dmx like 2)	612186
DFNB37 , DFNA22	*MYO6* (myosin VI)	600970
DIAPH3	*AUNA1* (diaphanous related formin 3)	614567
**Non-syndromic hearing loss human genes in the auditory hair cells**		
**Hair cell's adhesion & maintenance**		
DFNB29	*CLDN14* (claudin 14)	605608
DFNA51	*TJP2* (tight junction protein 2)	607709
DFNB42	*ILDR1* (immunoglobulin like domain containing receptor 1)	609739
DFNA15	*POU4F3* (POU class 4 homeobox 3)	602460
DFNB61	*SLC26A5* (solute carrier family 26 member 5)	604943
DFNB49	*MARVELD2* MARVEL domain containing 2 ()	610572
DFNA4A	*MYH14* (myosin heavy chain 14)	608568
DFNA17	*MYH9* (myosin heavy chain 9)	160775
DFNB76	*SYNE4* (spectrin repeat containing nuclear envelope family member 4)	615535
DFNB91	*SERPINB6* (serpin family B member 6)	173321
DFNA2A	*KCNQ4* (potassium voltage-gated channel subfamily Q member 4)	603537
DFNB109	*ESRP1* (epithelial splicing regulatory protein 1)	612959
DFNB111	*MPZL2* (myelin protein zero like 2)	604873
DFNA7	*LMX1A* (LIM homeobox transcription factor 1 alpha)	600298
DFNA75	*TRRAP* (transformation/transcription domain associated protein)	603015
**Non-syndromic hearing loss human genes in diverse inner ear cell types**		
**Cochlea ion homeostasis**		
DFNA3, DFNB1	*GJB2* (gap junction protein beta 2)	121011
DFNA2B	*GJB3* (gap junction protein beta 3)	603324
DFNA3B	*GJB6* (gap junction protein beta6)	604418
DFNA2A	*KCNQ4* (potassium voltage-gated channel subfamily Q member 4)	603537
DFNB4	*PDS,SLC26A4* (solute carrier family 26 member 4)	605646
DFNB8/10	*TMPRSS3* (transmembrane serine protease 3)	605511
DFNB60	*SLC22A4* (solute carrier family 22 member 4)	604190
DFNB68	*S1PR2* (sphingosine-1-phosphate receptor 2)	605111
DFNB73	*BSND* (barttin CLCNK type accessory subunit beta)	606412
DFNB103	*CLIC5* (chloride intracellular channel 5)	607293
DFNB115	*SPNS2* (sphingolipid transporter 2)	612584
DFNA41	*P2RX2* (purinergic receptor P2X 2)	600844
DFNAi	*SLC12A2 (*solute carrier family 12 member 2*)*	600840
**Non-syndromic hearing loss human genes in diverse inner ear cell types**		
**Transmembrane or secreted proteins<br>& Extracellular matrix**		
DFNB21, DFNA8	*12TECTA* (tectorin alpha)	602574
DFNB22	*OTOA* (otoancorin)	607038
DFNB18B	*OTOG* (otogelin)	604487
DFNB84B	*OTOGL* (otogelin like)	614925
DFNA4B,DFNB113	*CEACAM16* (CEA cell adhesion molecule 16, tectorial membrane component)	614591
DFNB26	*GAB1* (GRB2 associated binding protein 1)	604439
DFNB39	*HGF* (hepatocyte growth factor)	142409
DFNB97	*MET* (MET proto-oncogene, receptor tyrosine kinase)	164860

DFNA9, DFNB110	*COCH* (cochlin)	603196
DFNA13, DFNB53	*COL11A2* (collagen type XI alpha 2 chain)	120290
DFNA37	*COL11A1* (collagen type XI alpha 1 chain)	120280
DFNX6	*COL4A6* (collagen type IV alpha 6 chain)	303631
DFNA44	*CCDC50* (coiled-coil domain containing 50)	611051
DFNA56	*TNC* (tenascin C)	187380
DFNA66	*CD164* (CD164 molecule)	603356
DFNA69	*KITLG* (KIT ligand)	184745
**Non-syndromic hearing loss human genes in diverse inner ear cell types**		
**Oxidative stress, metabolism & mitochondria**		
DFNA5	*GSDME* (gasdermin E)	608798
DFNA6	*WFS1* (wolframin ER transmembrane glycoprotein)	606201
DFNA40	*CRYM* (thiomorpholine-carboxylate dehydrogenase)	123740
DFNA64	*DIABLO* (diablo IAP-binding mitochondrial protein)	605219
DFNA2C	*IFNLR1* (interferon lambda receptor1)	607404
DFNA74	*PDE1C* (phosphodiesterase 1C)	602987
DFNA70	*MCM2* (minichromosome maintenance complex component2)	116945
DFNA34	*NLRP3* (NLR family pyrin domain containing3)	606416
DFNX1	*PRPS1* (phosphoribosyl pyrophosphate synthetase 1)	311850
DFNB59	*PJVK* (pejvakin)	610219
DFNB89	*KARS1* (lysyl-tRNA synthetase 1)	601421
DFNB94	*NARS2* (asparaginyl-tRNA synthetase 2,mitochondria)	612803
DFNB74	*MSRB3* (methionine sulfoxide reductase B3)	613719
DFNB70	*PNPT1* (polyribonucleotide nucleotidyltransferase 1)	610316
DFNAi	*SCD5* (stearoyl-CoA desaturase 5)	608370
DFN mitochondrial	*MTRNR1* (RIBOSOMAL RNA, MITOCHONDRIAL, 12S; MTRNR1)	561000
DFN mitochondrial	*MTTS1* (tRNA-Ser TRANSFER RNA, MITOCHONDRIAL)	590080
**Non-syndromic hearing loss human genes in diverse inner ear cell types**		
**Transcriptional regulation**		
DFNA10	*EYA4* (EYA transcriptional coactivator and phosphatase 4)	603550
DFNA23	*SIX1* (SIX homeobox 1)	601205
DFNX2	*POU3F4* (POU class 3 homeobox 4)	300039
DFNB35	*ESRRB* (estrogen related receptor beta)	602167
DFNA28	*GRHL2* (grainyhead like transcription factor 2)	608576
DFNB49	*BDP1* (MARVEL domain containing 2)	610572

Table S2. Comparison of different routes of delivery to the inner ear.

Direct administration to the inner ear			
	**Posterior semicircular canal (PSCC)**		**Utricle**
Pros:	- wide delivery route through the labyrinth<br>- minimal manipulation of the temporal bone	Pros:	- minimal risk of distrupting the cochlea<br>- no hearing or balance damage<br>- easy access to the endolymphatic spaces
Cons:	- no risk of vestibular or auditory damage<br>- low auditory hair cells transduction in the adult ear	Cons:	- broad viral distribution & high efficient transduction<br>Cons: - not easily accessible for clinical application
	**Round window membrane (RWM)**		**Cochleostomy**
Pros:	- low risk of hearing damage<br>- safe & feasible delivery into perilymphatic space<br>- cinically used for cochlear implantation	Pros:	- direct access to the hearing sensory organ
Cons:	- limited viral transduction from base to apex<br>- inability to access endolymphatic spaces	Cons:	- too invasive<br>- Low volume for injections
	**Systemic administration**		**Middle ear administration**
Pros:	- oral dosage forms<br>- injectable solutions<br>- nanomedicines<br>- no harm to the inner ear	Pros:	- solutions and suspensions<br>- hydrogels<br>- nanomedicines<br>- medical devices
Cons:	- blood cochlear barrier<br>- low local vascularisation,<br>- diffusion side effects	Cons:	- limited crossing of round & oval windows<br>- clearance through Eustachian tube

Table S3. Adeno-associated virus (AAV) vectors used in inner ear gene therapy studies.

AAV vector	Transgene	Animal model	Route of delivery; age at delivery	Target cells/ outcomes	References
BAAV-CMV-β-actin -GFP	β-actin	Wild-type and deafened guinea pig	Cochleostomy and RMW; adult	Transduction of supporting cells, but no sensory hair cells.	Shibata et al, 2009
AAV2/1-CMV-eGFP (also AAV2/2, 2/5, 2/6, 2/8)	*eGFP*	Wild-type and deafened (kanamycin and furosemide injected) mice	Cochleostomy; 2-12 months	Efficient AAV inoculation in adult mouse ears, high transduction efficiency of IHCs, especially for serotypes 2 and 8, with hearing preservation.	Kilpatrick et al, 2011
AAV1-CBA-Kcnq1-eGFP	*Kcnq1*	*Kcnq1*$^{-/-}$ mice	Cochleostomy and RWM; P0-P2	High transduction of marginal cells, normal endocochlear potential and preserved cochlear morphology. Hearing improvement from 20 dB to complete rescue of the deafness phenotype at both low and high frequencies up to 18 weeks after treatment.	Chang et al, 2015
AAV2/1-CAG-eGFP (also AAV2/2, 5, 6, 6.2, 7, 8, 9, rh.8, rh.10, rh.39, rh.43)	*eGFP*	Wild-type mice	Cochleostomy; 6 weeks	AAV2/1, 2, 6.2, 7, 8, 9, rh.39, and rh.43 transduce IHCs, but not OHCs, with even partial OHC loss.	Shu et al, 2016
Anc80L65-CMV-WRPE-eGFP	*eGFP*	Wild-type mice	RWM; P0-P2	Anc80L65 round window membrane injection was well tolerated, as indicated by sensory cell function, hearing and vestibular function, and immunologic parameters. The ability of Anc80L65 to target OHCs at high rates, a requirement for complete restoration of auditory function, enables future gene therapies for hearing and balance disorders.	Landegger et al, 2017
AAV2/Anc80L65-CASI-eGFP-RBG	*eGFP*	Wild-type mice	PSCC; 7 weeks	Cochlea: Successful transduction of all IHCs, a majority of OHCs especially at the apex, and 10% of spiral ganglion neurons.<br>Vestibular end-organs: Robust transduction of macula and crista hair cells, and all supporting cells.	Suzuki et al, 2017
AAV2/1-CAG-eGFP (also AAV2/2, 6.2, 8, 9, rh.39, rh.43)	*eGFP*	Wild-type mice	PSCC; 8-10 weeks	Most AAVs transduced adult IHCs efficiently, but were less efficient at transducing OHCs. Subset of AAVs transduced other cell types.<br>Canalostomy can be a viable delivery route.	Tao et al, 2018

AAV2/Anc80L65-CMV-eGFP-WPRE					
AAV2/9-CMV-eGFP<br>AAV2/Anc80L65-CMV-eGFP	*eGFP*	Wild-type mice	RWM and RWM+ canal fenestration;<br>P15–16,<br>P56–60	Cochlea: RWM injection with AAV2/9: limited IHC transduction in basal turn with auditory threshold shift.<br>RWM + CF injection with AAV2/9: robust transgene expression without auditory threshold shift. Transduction efficiency in IHCs is independent of injection time point. Cochlear transduction: dose- and AAV serotype-dependent, but not age-dependent.<br>AAV2/Anc80L65 demonstrated superior transduction to AAV2/9. Robust eGFP transduction of all IHCs throughout the cochlea. Vestibular end-organs: Successful transduction with either posterior or lateral semi-circular canal fenestration.<br>AAV2/Anc80L65 transduction was superior to AAV2/9.	Yoshimura et al, 2018
AAV2/2-CBA-eGFP.<br>AAV2/9-CBA-eGFP<br>AAV2/Anc80L65-CMV- -globin-eGFP	*eGFP*	Wild-type mice	Cochleostomy;<br>P2-P3	AAV2/2-CBA: Few eGFP-positive sensory hair cells or supporting cells.<br>AAV2/Anc80L65-CMV: High transduction efficiency of both IHCs and OHCs for all cochlear turns. A tonotopic gradient for the transduction of supporting cells, with more supporting cells expressing eGFP at the apex than the base.<br>AAV2/9-CBA: A tonotopic gradient for the transduction of IHCs, with more IHCs expressing eGFP at the base than the apex, while the average transduction efficiency in OHCs was about 15% in all cochlear turns. The transduction efficiencies in supporting cells was similar to AAV2/Anc80L65-CMV.	Gu et al, 2019
AAV2/8-eGFP	*eGFP*	Wild-type mice	PSCC;<br>5-6 weeks	Canalostomy is an effective and safe approach for drug delivery into the inner ears of adult mice.	Guo et al, 2018
AAV2.7m8-CAG-eGFP	*eGFP*	Wild-type mice	PSCC;<br>P0-P5, 1-6 months	Cochlea: Highly efficient transduction of IHCs, OHCs, and a subset of leucine-rich repeat-containing G-protein coupled receptor 5 (LGR5)-positive supporting cells (inner pillar cells and inner phalangeal cells).<br>Vestibular end-organs: Only data for neonatal animals – less efficient transduction in vestibular organs than cochlea.	Isgrig et al, 2019

Anc80L65-CMV-eGFP<br>AAV9-PHP.B-CMV-eGFP<br>AAV2.7m8-CMV-eGFP-WPRE	*eGFP*	Wild-type mice	Utricle; RWM;<br>P1, P7, and P16	Cochlea: P1 utricle injection of AAV9-PHP.B-Cmv-eGFP: 80-100% of sensory hair cells transduced in all tonotopic regions of the cochlea.<br>Anc80L65 and AAV2.7m8 injections had lower efficiencies and more variable transduction in both IHCs and OHCs in all regions of the cochlea.<br>P7 or P16 utricle injection of AAV9-PHP.B-CMV-eGFP: eGFP expression in 100% of IHCs at both ages. 80 to 100% of OHCs were also GFP-positive at P7, with a lower efficiency at P16.<br>No alteration in auditory function (normal ABR and DPOAE thresholds for any frequency).<br>Vestibular end-organs: Robust transduction of vestibular hair cells at all time points tested for AAV9-PHP.B-CMV-eGFP injected mice.<br>No alteration in vestibular function (normal VsEP thresholds).	Lee et al, 2020
AAV9-PHP.B-CBA-eGFP-WPRE	*eGFP*	Cynomolgus monkeys	RWM;<br>1.5-5 years	100% transduction of both IHCs and OHCs, from base to apex, at the higher doses. It can be considered as a promising AAV for inner gene therapy in humans.	Ivanchenko et al, 2020

BAAV, Bovine AAV; CMV, cytomegalovirus enhancer; RWM, round window membrane; eGFP, *enhanced green fluorescent protein*; PSCC, posterior semicircular canal; IHCs, inner hair cells; OHCs, outer hair cells; CBA, chicken β-actin; WPRE, woodchuck hepatitis virus post-transcriptional regulatory element; CASI, CMV enhancer fused to chicken β-actin promoter, and UBC (Ubiquitin C) enhancer as well as splice donor and acceptor sequences; CAG, CMV enhancer fused to the chicken β-actin promoter; ABR, auditory-evoked brainstem response; DPOAE: Distortion product otoacoustic emissions; VsEP, vestibular evoked potential.
